# Intake of B vitamins and the risk of developing islet autoimmunity and type 1 diabetes in the TEDDY study

**DOI:** 10.1007/s00394-024-03346-6

**Published:** 2024-02-27

**Authors:** Leena Hakola, Lazarus K. Mramba, Ulla Uusitalo, Carin Andrén Aronsson, Sandra Hummel, Sari Niinistö, Iris Erlund, Jimin Yang, Marian J. Rewers, Beena Akolkar, Richard A. McIndoe, Stephen S. Rich, William A. Hagopian, Anette Ziegler, Åke Lernmark, Jorma Toppari, Jeffrey P. Krischer, Jill M. Norris, Suvi M. Virtanen, The TEDDY Study Group, Marian Rewers, Kimberly Bautista, Judith Baxter, Daniel Felipe-Morales, Brigitte I Frohnert, Marisa Stahl, Isabel Flores Garcia, Patricia Gesualdo, Sierra Hays, Michelle Hoffman, Randi Johnson, Rachel Karban, Edwin Liu, Leila Loaiza, Jill Norris, Holly O’Donnell, Loana Thorndahl, Andrea Steck, Kathleen Waugh, Olli G Simell, Annika Adamsson, Suvi Ahonen, Mari Åkerlund, Sirpa Anttila, Anne Hekkala, Tiia Honkanen, Teija Hurskainen, Heikki Hyöty, Jorma Ilonen, Saori Itoshima, Minna Jokipolvi, Sanna Jokipuu, Taru Karjalainen, Leena Karlsson, Jukka Kero, Marika Korpela, Jaakko J Koskenniemi, Miia Kähönen, Mikael Knip, Minna-Liisa Koivikko, Katja Kokkonen, Merja Koskinen, Mirva Koreasalo, Kalle Kurppa, Salla Kuusela, Jarita Kytölä, Mia Laakso, Jutta Laiho, Tiina Latva-aho, Siiri Leisku, Laura Leppänen, Katri Lindfors, Maria Lönnrot, Elina Mäntymäki, Markus Mattila, Maija Miettinen, Tiina Niininen, Noora Nurminen, Sami Oikarinen, Hanna-Leena Oinas, Paula Ollikainen, Zhian Othmani, Sirpa Pohjola, Solja Raja-Hanhela, Jenna Rautanen, Anne Riikonen, Minna Romo, Juulia Rönkä, Nelli Rönkä, Satu Simell, Aino Tihinen, Päivi Tossavainen, Mari Vähä-Mäkilä, Eeva Varjonen, Riitta Veijola, Irene Viinikangas, Silja Vilmi, Suvi M Virtanen, Richard McIndoe, Desmond Schatz, Diane Hopkins, Michael Haller, Melissa Gardiner, Ashok Sharma, Laura Jacobsen, Percy Gordon, Jennifer Hosford, Sharon Maina, Chelsea Salmon, Anette G Ziegler, Ezio Bonifacio, Cigdem Gezginci, Willi Grätz, Anja Heublein, Annette Knopff, Sibylle Koletzko, Claudia Ramminger, Roswith Roth, Jennifer Schmidt, Marlon Scholz, Joanna Stock, Katharina Warncke, Lorena Wendel, Christiane Winkler, Daniel Agardh, Rasmus Bennet, Corrado Cilio, Susanne Dahlberg, Malin Goldman Tsubarah, Emelie Ericson-Hallström, Lina Fransson, Emina Halilovic, Susanne Hyberg, Berglind Jonsdottir, Naghmeh Karimi, Helena Elding Larsson, Marielle Lindström, Markus Lundgren, Marlena Maziarz, Jessica Melin, Kobra Rahmati, Anita Ramelius, Falastin Salami, Anette Sjöberg, Evelyn Tekum Amboh, Carina Törn, William A Hagopian, Michael Killian, Claire Cowen Crouch, Jennifer Skidmore, Trevor Bender, Megan Llewellyn, Cody McCall, Arlene Meyer, Jocelyn Meyer, Denise Mulenga, Nole Powell, Jared Radtke, Shreya Roy, Preston Tucker, Dorothy Becker, Margaret Franciscus, MaryEllen Dalmagro-Elias Smith, Ashi Daftary, Mary Beth Klein, Chrystal Yates, Jeffrey P Krischer, Rajesh Adusumali, Sarah Austin-Gonzalez, Maryouri Avendano, Sandra Baethke, Brant Burkhardt, Martha Butterworth, Nicholas Cadigan, Joanna Clasen, Kevin Counts, Laura Gandolfo, Jennifer Garmeson, Veena Gowda, Christina Karges, Shu Liu, Xiang Liu, Kristian Lynch, Jamie Malloy, Lazarus Mramba, Cristina McCarthy, Jose Moreno, Hemang M Parikh, Cassandra Remedios, Chris Shaffer, Susan Smith, Noah Sulman, Roy Tamura, Dena Tewey, Henri Thuma, Michael Toth, Kendra Vehik, Ponni Vijayakandipan, Melissa Wroble, Kenneth Young, Liping Yu, Dongmei Miao, Kathleen Gillespie, Kyla Chandler, Olivia Pearce, Sarah Stollery, Elinor Balch, Hanah Batholomew, Zahra Hashmi, William Hagopian, Jared Radtke, Preston Tucker, Thomas Briese, Todd Brusko, Teresa Buckner, Suzanne Bennett Johnson, Eoin McKinney, Tomi Pastinen, Steffen Ullitz Thorsen, Eric Triplett

**Affiliations:** 1 Faculty of Social Sciences, Unit of Health Sciences, Tampere University, 33014 Tampere, Finland; 2 Tampere University Hospital, Wellbeing Services County of Pirkanmaa, Tampere, Finland; 3 Health Informatics Institute, Morsani College of Medicine, University of South Florida, Tampa, FL USA; 4 Department of Clinical Sciences, Lund University, Malmö, Sweden; 5 Pediatric department, Skåne University Hospital, Malmö, Sweden; 6 Institute of Diabetes Research, Helmholtz Munich, German Research Center for Environmental Health, Munich, Germany; 7 Forschergruppe Diabetes E.V.at Helmholtz Zentrum München, Munich, Germany; 8 School of Medicine, Technical University Munich, Forschergruppe Diabetes at Klinikum Rechts Der Isar, Munich, Germany; 9 Health and Well-Being Promotion Unit, Finnish Institute for Health and Welfare, Helsinki, Finland; 10 Department of Government Services, Finnish Institute for Health and Welfare, Helsinki, Finland; 11 Davis Center for Childhood Diabetes, University of Colorado, Aurora, CO USA; 12 National Institute of Diabetes and Digestive and Kidney Diseases, Bethesda, MD USA; 13 Center for Biotechnology and Genomic Medicine, Medical College of Georgia, Augusta University, Augusta, GA USA; 14 Center for Public Health Genomics, University of Virginia, Charlottesville, VA USA; 15 Pacific Northwest Research Institute, Seattle, WA USA; 16 Klinikum Rechts Der Isar, Forschergruppe Diabetes E.V, Technische Universität München, Neuherberg, Germany; 17 Department of Clinical Sciences, Lund University CRC, Skåne University Hospital, Malmö, Sweden; 18 Department of Pediatrics, Turku University Hospital, Turku, Finland; 19 Institute of Biomedicine, Research Centre for Integrative Physiology and Pharmacology, and Centre for Population Health Research, University of Turku, Turku, Finland; 20 Department of Epidemiology, Colorado School of Public Health, University of Colorado Anschutz Medical Campus, Aurora, CO 80045 USA; 21 Center for Child Health Research, Tampere University and Tampere University Hospital, Tampere, Finland

**Keywords:** Diabetes mellitus, type 1, Birth cohort, Nutrient intake, Vitamin B complex

## Abstract

**Purpose:**

The aim was to study the association between dietary intake of B vitamins in childhood and the risk of islet autoimmunity (IA) and progression to type 1 diabetes (T1D) by the age of 10 years.

**Methods:**

We followed 8500 T1D-susceptible children born in the U.S., Finland, Sweden, and Germany in 2004 -2010 from the Environmental Determinants of Diabetes in the Young (TEDDY) study, which is a prospective observational birth cohort. Dietary intake of seven B vitamins was calculated from foods and dietary supplements based on 24-h recall at 3 months and 3-day food records collected regularly from 6 months to 10 years of age. Cox proportional hazard models were adjusted for energy, HLA-genotype, first-degree relative with T1D, sex, and country.

**Results:**

A total of 778 (9.2) children developed at least one autoantibody (any IA), and 335 (3.9%) developed multiple autoantibodies. 280 (3.3%) children had IAA and 319 (3.8%) GADA as the first autoantibody. 344 (44%) children with IA progressed to T1D. We observed that higher intake of niacin was associated with a decreased risk of developing multiple autoantibodies (HR 0.95; 95% CI 0.92, 0.98) per 1 mg/1000 kcal in niacin intake. Higher intake of pyridoxine (HR 0.66; 95% CI 0.46, 0.96) and vitamin B12 (HR 0.87; 95% CI 0.77, 0.97) was associated with a decreased risk of IAA-first autoimmunity. Higher intake of riboflavin (HR 1.38; 95% CI 1.05, 1.80) was associated with an increased risk of GADA-first autoimmunity. There were no associations between any of the B vitamins and the outcomes “any IA” and progression from IA to T1D.

**Conclusion:**

In this multinational, prospective birth cohort of children with genetic susceptibility to T1D, we observed some direct and inverse associations between different B vitamins and risk of IA.

**Supplementary Information:**

The online version contains supplementary material available at 10.1007/s00394-024-03346-6.

## Introduction

Type 1 diabetes (T1D) is characterized by the appearance of circulating islet autoantibodies indicating islet autoimmunity (IA) before clinical diagnosis [1]. Both genetic and environmental factors contribute to the development of IA and T1D. It has been suggested that there might be endotypes of T1D, with specific first appearing autoantibodies and possibly endotype-specific risk factors [1].

Dietary factors as risk or protective factors of islet autoimmunity or T1D have been studied relatively widely, mostly in observational studies [2], however, the B vitamins have so far gained only little attention. The B vitamins are a group of water-soluble vitamins with a variety of functions in metabolism and immune response. For example, dietary folate or folic acid, pyridoxine, and vitamin B12 act in one-carbon metabolism; thiamin and riboflavin, and pantothenic acid are involved in energy metabolism, and niacin and riboflavin act in oxidation–reduction reactions [3]. A wide variety of foods, such as cereals, vegetables, dairy and meat contribute to the intake B vitamins with some differences between specific B vitamins [3].

In the U.S. Diabetes Autoimmunity Study in the Young (DAISY) Study, higher intake of niacin, vitamin B12, and riboflavin at seroconversion to islet autoantibody positivity was associated with a decreased risk of progression to T1D [4]. These vitamins were part of a dietary pattern that was linked to a metabolomic profile associated with progression to T1D [4]. Earlier, nicotinamide (a form a niacin) was found to be promising in prevention of T1D in mice [5] but showed no effect on progression to T1D in humans, in a randomized controlled 5-year trial where oral nicotinamide was given to participants in pharmacological doses [6]. In a Canadian case–control study the intake of six B vitamins (thiamin, riboflavin, niacin, pyridoxine, vitamin B12, and folate) a year before diagnosis did not differ between children and adolescents with T1D and their matched controls [7]. Thus, the current knowledge on B vitamins and risk of T1D is based on individual studies with various study designs. To our knowledge, there are no prospective studies that have consistently explored the associations between intake of B vitamins in childhood and risk of IA or T1D. However, due to the B vitamins’ multiple roles in physiology, a higher intake could theoretically protect from IA and T1D.

The aim of this study was to investigate the association between longitudinally assessed dietary intake of thiamin, riboflavin, niacin, pantothenic acid, pyridoxine, folate/folic acid, and cobalamin and the development of T1D related outcomes, IA and progression to T1D.

## Methods

### Subjects and methods

#### Study design and population

The Environmental Determinants of Diabetes in the Young (TEDDY) is a prospective observational birth cohort study designed to identify environmental triggers of T1D [8]. The enrollment of newborn children was carried out in clinical centers located in the USA (Washington, Colorado, and Georgia), Finland, Sweden, and Germany, from September 2004 through February 2010. The study includes children with genetic and familial susceptibility for T1D. Eligibility criteria for initial contact was one of the following HLA DR genotypes: HLA-DR3/4; HLA-DR4/4; HLA-DR4/8; HLA-DR3/3 and HLA-DR4/4. Infants with HLA-DR genotypes HLA-DR4/1, HLA-DR4/13, HLA-DR4/9 and HLA-DR3/9 were included only if they had a first-degree relative with T1D [9].

For all participants, written informed consent was obtained from a parent or primary caretaker for genetic screening, and separate consent was obtained for participation in the prospective follow-up. The study was approved by the local Institutional Review or Ethics Boards at each site and is monitored by an External Evaluation Committee formed by the National Institutes of Health.

Follow-up visits at the study clinics were scheduled every 3 months between ages 3 months to 48 months of age, and biannually thereafter. However, children with islet autoantibodies were followed up every 3 months regardless of the age. In the present study the follow-up ended at 10 years of age.

#### Vitamin B intake

Intake of seven B vitamins [thiamin (B1), riboflavin (B2), niacin (B3), pantothenic acid (B5), pyridoxine (B6), folate/folic acid (B9), and cobalamin (B12)] was calculated based on food and dietary supplement consumption. Foods included foods and drinks (including breastmilk) reported in a 24-h recall at 3 months of age and 3-day food records at 6-months to 10 years of age. The three-day food records were collected at the age of 6, 9, and 12 months, and biannually until the age of 10 years. Primary caretakers were trained during the 3-month clinic visit to keep 3-day food records of the child’s dietary intake. Participants were instructed to fill out the 3-day food record within 10 days prior to the next clinic visit. At each clinic visit, the diet records were reviewed by trained study personnel together with the primary caretaker. Amount of breastmilk was estimated based on the difference between estimated total energy expenditure [10] and energy intake from other foods and drinks among the breastfed children. Use of dietary supplements with B vitamins (yes/no) and daily intake of B vitamins from the supplements (mg/day or µg/day) was calculated was based on all dietary supplements listed to the 24-h recall and 3-day food records by the caretakers at each visit.

Description of the dietary assessment methods used in TEDDY has been described in detail before [11]. Food record data entry was done by trained personnel. Calculation of vitamin B intake was based on four national food composition databases: FINELI (Finland), LEBTAB (Germany), NFA Food Composition Database (Sweden), and Nutrition Data System for Research software developed by the University of Minnesota Nutrition Coordinating Center (NCC) (US). After the harmonization efforts of the food composition databases, TEDDY researchers concluded that intake of thiamin, riboflavin, niacin, pyridoxine, and cobalamin are comparable between all four countries [12]. Pantothenic acid is not available in the Swedish database, and therefore comparable only between three countries. Folate in foods has been analyzed using varying methods across the TEDDY countries and folate intake is not comparable across countries [12]. In calculation of total dietary folate equivalents from foods and supplements, the supplementary folic acid was multiplied with 1.7 to take higher bioavailability of folic acid in comparison to dietary folate into account [13].

All vitamin B intakes were treated as time-dependent exposure variables where the data was arranged using the counting process with risk sets in a longitudinal format, including all dietary assessments from 3 months to 10 years of age.

#### T1D related outcomes

Blood samples were obtained at each study visit to screen for islet autoantibodies against insulin (IAA), glutamic acid decarboxylase (GADA), and insulinoma antigen-2 (IA-2A) [8, 14]. “Any IA” was defined as any autoantibody positivity on two or more consecutive visits 3 months apart and confirmed at two laboratories. Multiple IAs was defined as repeated positivity to at least two autoantibodies. In addition, we studied IAA and GADA as first appearing autoantibodies, and progression from antibody positivity to T1D. T1D was defined using American Diabetes Association criteria [15].

#### Statistical methods

Data analyses involved examining associations between intake of seven B vitamins with the development of T1D related outcomes (persistent IA, IAA first, GADA first, multiple IA and progression from IA to T1D) during the first 10 years of life.

Each vitamin intake was adjusted for energy using the multivariable nutrient density method [16, 17] that includes dividing the vitamin intake with total energy intake and taking the energy as a covariate in the model. Outliers were detected and set to missing using the interquartile range method [18] with a scale factor of 5. (Cause-specific) Cox proportional hazard models were used to analyze the associations of these time-dependent exposure variables and the risk of each outcome, adjusted for energy as mentioned, as well as for HLA-genotype (DR3/4, no DR3/4), first-degree relative with T1D (yes, no), sex (female, male), and country (U.S., Finland, Germany, Sweden) and displayed using hazard ratios (HR) with 95% confidence intervals (CI). Covariates were selected based on previous observations in TEDDY study [1] and study question-based consideration. Participants were followed until they got the outcome, or they were censored at their last visit, or age of 10 years, whichever came first. Loss to follow-up was considered a random event with no additional information provided in our database. Analyses related to pantothenic acid was performed excluding Sweden because data was not available from this country. Intake of folate/folic acid was not comparable among countries. Thus, the analyses related to folate/folic acid were performed separately for each country. Cox proportional hazard models were used to analyze the association of vitamin intake at the time of seroconversion with the risk of progression from IA to T1D with the adjustment for energy, HLA-genotype, first-degree relative with T1D, sex, country, age at seroconversion, and first autoantibody at seroconversion (IAA only, GADA only, IA-2A only or multiple antibodies). Robust standard errors were used to account for within subject correlations. The shape of associations for those with p < 0.05 in the main analyses was assessed with restricted cubic splines.

To assess, whether conditions that could affect B vitamin absorption or metabolism, could lead to misclassification of the exposure and mask the associations between B vitamin intake and IA, we performed a sensitivity analyses for “any IA” outcome by excluding 603 children with autoimmune hepatitis (n = 1), autoimmune thyroiditis (n = 49), Crohn's disease (n = 1), epilepsy and recurrent seizures (n = 3), intestinal malabsorption (n = 11), and celiac disease (n = 538).

Interaction terms were included in the models to evaluate whether sex, first-degree relative, HLA-genotype or country modified the associations between vitamin intake and each of the T1D related outcomes. Folate was not included in the interaction analyses because of the limited number of participants per country.

The results were not adjusted for multiple comparisons because all exposure variables were selected a priori. Data analysis was carried out using the Statistical Analysis System Software SAS® Software 9.4 (SAS/STAT 15.2) and R Core Team (2023).

## Results

Of the 8676 children enrolled in the study, 8500 subjects were included in the analyses (Supplementary Fig. 1). The TEDDY cohort has been described previously [14] and summarized in Table 1. During the 10 years of follow-up, 778 of the 8500 participants (9.2%) developed persistent confirmed IA with a median (IQR) age of 36 (18, 73) months. A total of 280 (3.3%) participants had IAA as the first appearing autoantibody at median (IQR) age of 22 (12, 45) months, 319 (3.8%) participants had GADA as the first appearing autoantibody at median (IQR) age of 50 (27, 86) months. A total of 344 (44%) of 788 children with IA children progressed from IA to T1D at the median (IQR) age of 82 (43, 118) months, 45 (18, 75) months after first seroconversion. The median (IQR) number of nutrient assessments per child was 9 (4, 18).

**Table 1:** Characteristics of TEDDY study population

	Total	IA	IAA-first	GADA-first	Multiple IA	Progression from IA to T1D
	n	n (% of total)	n (% of total)	n (% of total)	n (% of total)	n (% of IA)
	8500	778 (9.2)	280 (3.3)	319 (3.8)	335 (3.9)	344 (44.2)
Country						
U.S.	3615	265 (7.3)	89 (2.5)	117 (3.2)	110 (3.0)	115 (43.4)
Finland	1803	195 (10.8)	90 (5.0)	68 (3.8)	87 (4.8)	99 (50.8)
Germany	580	57 (9.8)	18 (3.1)	15 (2.6)	26 (4.5)	35 (61.4)
Sweden	2502	261 (10.4)	83 (3.2)	119 (4.8)	112 (4.5)	95 (36.4)
First-degree relative with T1D						
Yes	961	155 (16.1)	62 (6.5)	54 (5.6)	79 (8.2)	92 (59.4)
No	7539	623 (8.3)	218 (2.9)	265 (3.5)	256 (3.4)	252 (40.4)
Sex						
Female	4196	351 (8.4)	124 (3.0)	151 (3.6)	141 (3.4)	156 (44.4)
Male	4304	427 (9.9)	156 (6.6)	168 (3.9)	194 (4.5)	188 (44.0)
HLA-DR3/4Genotype						
Yes	3323	380 (11.4)	136 (4.1)	157 (4.7)	185 (5.6)	190 (50.0)
No	5177	398 (7.7)	144 (2.8)	162 (3.1)	150 (2.9)	154 (38.7)

*IA*, persistent islet autoimmunity, *IAA*-*first* IA with IAA as first antibody, *GADA*-*first* IA with GADA as first antibody, *T1D* type 1 diabetes

First-degree relative (mother, father, and/or sibling) with T1D

**Fig. 1:**
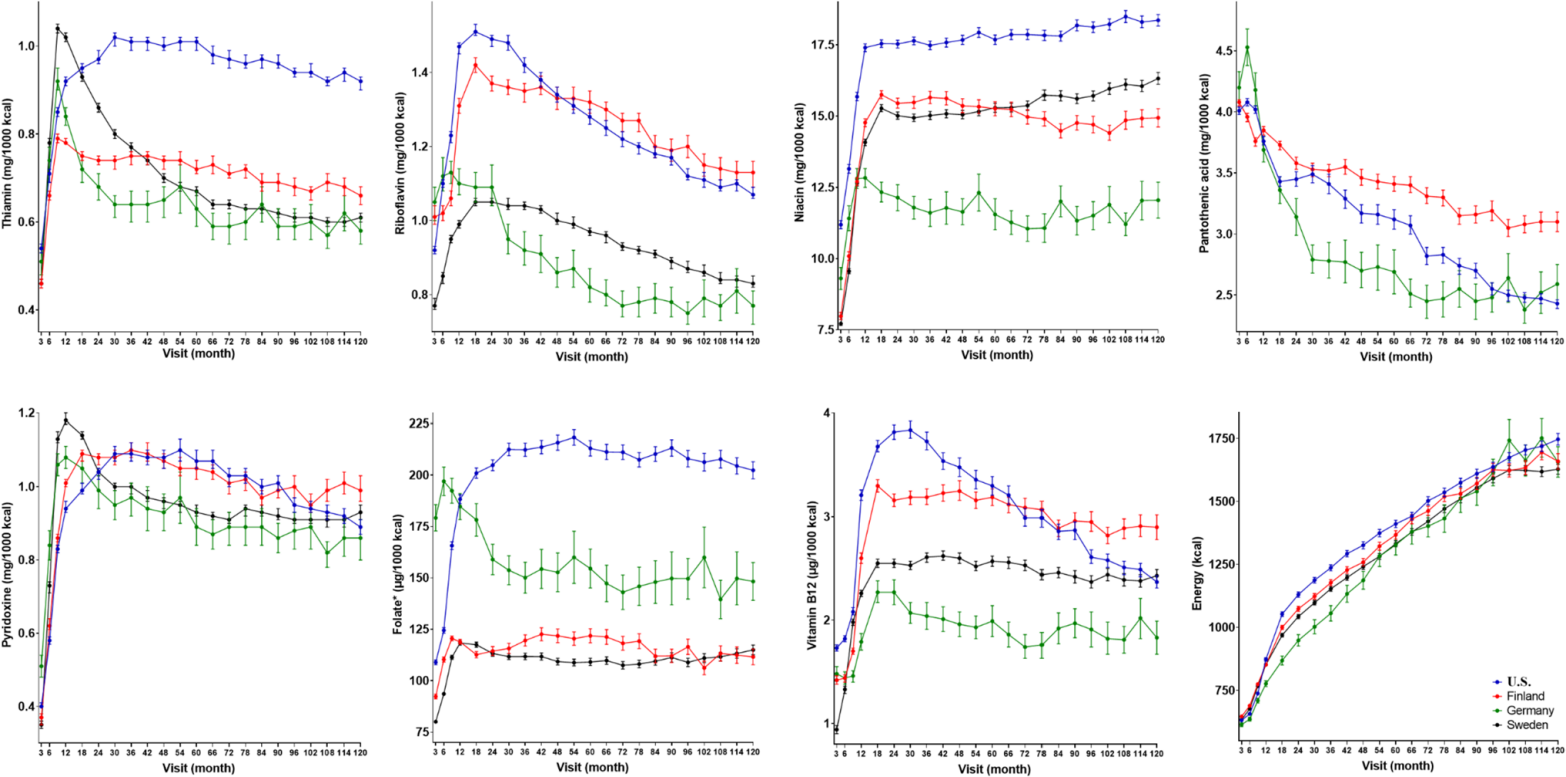
Mean (95% CI) intake of B vitamins per 1000 kcal of energy intake, and energy intake by visit and country in TEDDY children. *Folate values are not comparable between the countries

Intake of energy and B vitamins by age and country are presented in Fig. 1. The proportion of children using dietary supplements that contained B vitamins varied by age and by vitamin in question. The median proportion of B vitamin supplement users of all ages and vitamins was 5.2%. Intake from supplements was most common for pantothenic acid at the age of 5.5 years when 14.5% of the children had pantothenic acid intake from supplements. B vitamin supplementation was least common at the age of 3 months when proportion of children having B vitamins from supplements was < 0.5% for each vitamin.

The baseline covariate-adjusted and energy-adjusted vitamin B intake from foods and supplements were studied as continuous variables in relation to the T1D related outcomes and were considered the main analyses (Table 2). There was no association between any of the B vitamins and risk of developing “any IA” (consequent positivity to any islet autoantibody) nor the risk of progressing to T1D (Table 2). However, we observed that higher intake of niacin was associated with a decreased risk of developing multiple autoantibodies (Table 2). Higher intake of pyridoxine and vitamin B12 was associated with a decreased risk of IAA-first autoimmunity. Higher intake of riboflavin was associated with increased risk of GADA-first autoimmunity (Table 2). The shapes of the associations with p < 0.05 are presented in Supplementary Fig. 2.

**Table 2:** Associations between intake of B vitamins and risk of type 1 diabetes (T1D)-related outcomes in children aged 0 to 10 years, the TEDDY study

n outcome/cohort	Any IA		IAA-first		GADA-first		Multiple IA		Progression from IA to T1D	
	778/8500		280/8500		319/8500		335/8500		344/778	
	HR (95% CI)^a^	p	HR (95% CI)^a^	p	HR (95% CI)^a^	p	HR (95% CI)^a^	p	HR (95% CI)^a^	p
Thiamin (mg/1000 kcal)	0.97 (0.72, 1.31)	0.857	0.91 (0.60, 1.40)	0.669	1.19 (0.78, 1.82)	0.424	0.69 (0.44, 1.06)	0.091	0.95 (0.62, 1.43)	0.794
Riboflavin (mg/1000 kcal)	1.01 (0.83, 1.24)	0.901	0.77 (0.55, 1.06)	0.112	**1.38 (1.05, 1.80)**	**0.020**	0.99 (0.75, 1.30)	0.917	0.91 (0.64, 1.29)^3^	0.591
Niacin (mg/1000 kcal)	0.99 (0.96, 1.01)	0.198	0.98 (0.94, 1.01)	0.233	1.01 (0.97, 1.05)	0.608	**0.95 (0.92, 0.98)**	**0.003**	1.00 (0.96, 1.03)	0.779
Pantothenic acid (mg/1000 kcal) ^b^	1.00 (0.93, 1.08)	0.926	0.95 (0.83, 1.08)	0.421	1.05 (0.94, 1.16)^c^	0.398	1.05 (0.95, 1.15)	0.344	0.99 (0.87, 1.11)	0.820
Pyridoxine (mg/1000 kcal)	0.81 (0.63, 1.04)	0.104	**0.66 (0.46, 0.96)**	**0.028**	0.96 (0.66, 1.40)^c^	0.831	0.80 (0.57, 1.11)	0.185	1.14 (0.84, 1.54)	0.413
Vitamin B12 (µg/1000 kcal)	0.95 (0.89, 1.02)	0.137	**0.87 (0.77, 0.97)**	**0.017**	0.99 (0.91, 1.08)	0.877	0.96 (0.88, 1.04)	0.327	0.95 (0.85, 1.05)	0.314
Folate (10 µg/1000 kcal)										
U.S	1.00 (0.98, 1.02)	0.780	0.99 (0.95, 1.02)	0.432	1.01 (0.99, 1.04)	0.417	0.97 (0.94, 1.00)	0.060	0.98 (0.94, 1.01)	0.212
Finland	1.00 (0.97, 1.04)	0.937	1.00 (0.95, 1.06)	0.972	1.01 (0.94, 1.08)	0.827	0.99 (0.94, 1.04)	0.642	1.04 (0.97, 1.11)	0.296
Germany	0.98 (0.93, 1.03)	0.363	1.01 (0.96, 1.06)	0.726	1.00 (0.93, 1.08)	0.986	0.97 (0.91, 1.03)	0.301	1.03 (0.94, 1.13)	0.571
Sweden	0.98 (0.94, 1.02)	0.225	0.96 (0.87, 1.05)	0.322	1.01 (0.97, 1.05)	0.699	1.00 (0.96, 1.04)	0.924	0.96 (0.89, 1.03)	0.263

Vitamin intake from foods and dietary supplements combined

*Any IA* islet autoantibody positivity on two consecutive visit, *IAA-first* IA with IAA as first antibody, *GADA*-*first* IA with GADA as first antibody, *T1D* type 1 diabetes

Bolding refers to *p* < 0.05

^a^HR (95% CI) are based on Cox regression models ran separately for each vitamin. HR for 1 unit (mg or µg/1000 kcal) increase in intake. For folate, per 10 µg/1000 kcal increase. Adjusted for energy intake, HLA-DR3/4 genotype, sex, first-degree relative with T1D, country (except for folate); progression analyses also adjusted for age at seroconversion and first autoantibody

^b^No pantothenic acid data available for Swedish children

^c^Stratified associations due to observed interaction presented in text

In a sensitivity analysis for “any IA”, we excluded children with chronic conditions, and again, observed no associations between B vitamin intake and “any IA”.

We observed some interactions between intake of individual B vitamins and background characteristics with GADA-first outcome or progression to T1D but not with other outcomes. The stratified associations were studied for those with interaction p < 0.05: Pantothenic acid intake was positively associated with GADA-first autoimmunity in children with a first-degree relative with T1D [HR 1.24 (95% CI 1.06, 1.44)] but not in children without a first-degree relative with T1D [HR 0.98 (95% CI 0.86, 1.12)], p for interaction = 0.017. Pyridoxine intake tended to decrease the risk of GADA-first autoimmunity among girls [HR 0.56 (95% CI 0.27, 1.15)] and increase it among boys [HR 1.34 (95% CI 0.88, 2.03)], p for interaction = 0.0496. Finally, the riboflavin intake was positively associated with risk of progression to T1D among Finnish children [HR 1.69 (95% CI 1.01, 2.85)], but the association was inverse among children from the U.S. [HR 0.62 (95% CI 0.36, 1.07)], Germany [HR 0.67 (95% CI 0.12, 3.80)], and Sweden [HR 0.29 (95% CI 0.12, 0.69)], p for interaction = 0.014.

## Discussion

In this prospective birth cohort with T1D susceptible children from four countries, intake of seven studied B vitamins was not consistently associated with IA or T1D. However, higher niacin intake was associated with a decreased risk of IA to multiple autoantibodies, and higher pyridoxine and vitamin B12 intake was associated with a decreased risk of IAA-first autoimmunity. Finally, higher riboflavin intake was associated with an increased risk of GADA-first autoimmunity.

Strengths of this study include a large multinational study population and prospective study design, the repeated 3-day food records conducted consistently over time across four countries, the harmonization of four national food composition databases [12], frequent and longitudinal follow-up of islet autoantibody assessments with identification of first-appearing islet autoantibodies. Although the food composition databases were harmonized, the countries had somewhat different vitamin intake profiles. However, we were able to adjust for the country and study potential country-vitamin intake interactions with the outcomes. To our knowledge, this is the first prospective cohort to report association of several B vitamins with risk the of IA. A limitation is that we assessed only the *intake* of B vitamins, while not considering other factors that are related to vitamin absorption, metabolism, and availability. However, exclusion of children with conditions potentially affecting the utilization of B vitamins, such as coeliac disease, did not change the results regarding “any IA”. Another limitation is that the study is observational and no conclusions on causality can be made. The observed associations may be confounded by unknown factors, including but not limited to other components if foods rich in B vitamins. As our study population comprised of children with increased risk of type T1D, the findings may not apply to general population. However, the country did not modify most of the studied associations suggesting that our findings may be generalized to high-risk children in Northern and Central Europe, and the U.S.

We observed that higher intake of niacin was associated with a decreased risk of autoimmunity to multiple autoantibodies. Our finding is similar to a finding in the DAISY study, that niacin intake was inversely associated with the progression to T1D both as part of a dietary pattern and on its own [4], and in line with the old hypotheses suggesting that niacin’s derivate nicotinamide could prevent T1D [5, 6]. Niacin’s inverse association with developing multiple autoantibodies could be due to niacin’s antioxidant properties. In diabetes prone mice, niacin’s component nicotinamide prevented diabetes and beta-cell damage [5] perhaps by antioxidant mechanisms. Also another antioxidant, vitamin C assessed as plasma ascorbic acid has shown an inverse association with islet autoimmunity in the TEDDY study [19].

We observed that higher intake of pyridoxine and vitamin B12 was associated with a decreased risk of IAA-first autoimmunity. To our knowledge, pyridoxine has not been linked to any T1D-related outcome before but vitamin B12 intake was associated with a decreased risk of progression to T1D in the DAISY study [4]. The mechanisms between the inverse association of pyridoxine and vitamin B12 intake with IAA-first autoimmunity is not known. However, these vitamins are involved in immune regulation [20] and therefore may affect the risk of IA via immune modulation. Additionally, higher maternal blood concentrations of B12 have been linked to lower insulin resistance in children [21] suggesting a potential link, and pyridoxine might act via γ-aminobutyric acid metabolism [22].

Our observation that higher intake of riboflavin was associated with an increased risk of GADA-first autoimmunity differs from an inverse association between riboflavin intake and the progression to T1D observed in the DAISY study [4]. However, we observed country-interaction: and an inverse association between riboflavin intake and progression to T1D was seen among children in Sweden, and a direct association between riboflavin intake and progression to T1D among children in Finland. One possibility to explain the associations between riboflavin and risk of T1D-related outcomes is that cow’s milk, which has been linked with the increased risk of IA in other studies [23, 24] is a typical dietary source of riboflavin. Therefore, the riboflavin-GADA-first association in the whole cohort, and the riboflavin-T1D association in Finnish children may be confounded by some other compound in cow’s milk. The differences between countries could be explained by different food sources of riboflavin, and therefore reflect something else than riboflavin intake as such. For example, in Finland, dairy products contribute to major part (about 40%) of the riboflavin intake [25], while dairy’s proportion as riboflavin’s source is about 30% in Sweden [26] and about 20% in the U.S. [13]. In the U.S. fortified cereals are an important source of riboflavin [13].

To conclude, dietary intake of B vitamins showed some, non-consistent associations with IA and, in general, no association with progression to T1D. Based on our findings and the limited number of previous studies niacin, and vitamin B12 are the most interesting B vitamins in the etiology of T1D. However, the observed associations are weak and not fully consistent, and need replication in other prospective cohorts.

### Supplementary Information

Below is the link to the electronic supplementary material.

Supplementary file1 (DOCX 393 KB)

## Data Availability

“Data from The Environmental Determinants of Diabetes in the Young (10.58020/y3jk-x087) reported here will be made available for request at the NIDDK Central Repository (NIDDK-CR) website, Resources for Research (R4R), https://repository.niddk.nih.gov/.”

## Abbreviations

DAISY: Diabetes Autoimmunity Study in the Young
GADA: Glutamic acid decarboxylase autoantibody
IA: Islet autoimmunity
IAA: Insulin autoantibody
IA-2A: Insulinoma antigen-2 autoantibody
T1D: Type 1 diabetes
TEDDY: The Environmental Determinants of Diabetes in the Young
